# Angiotensin II and Renal Tubular Ion Transport

**DOI:** 10.1100/tsw.2005.92

**Published:** 2005-08-29

**Authors:** Patricia Valles, Jan Wysocki, Daniel Batlle

**Affiliations:** ^1^ Area de Fisiopatología, Departamento de Patología, Facultad de Ciencias Médicas, Universidad Nacional de Cuyo, Mendoza, Argentina; ^2^ Division of Nephrology and Hypertension, Department of Medicine, Northwestern University, The Feinberg School of Medicine, Chicago, USA

**Keywords:** Angiotensin II, Bicarbonate transport, Vacuolar H^+^, ATPase, Proximal convoluted tubule (PCT) Cortical collecting ducts (CCDs), Medullary collecting ducts (MCDs), ATP6V_1 B1_, ATP6V_0_A4

## Abstract

Angiotensin II, a potent vasoconstrictor, also participates in the regulation of renal sodium and water excretion, not only via a myriad of effects on renal hemodynamics, glomerular filtration rate, and regulation of aldosterone secretion, but also via direct effects on renal tubule transport. In addition, angiotensin II stimulates H^+^ secretion and HCO_3_^–^ reabsorption in both proximal and distal tubules and regulates H^+^-ATPase activity in intercalated cells of the collecting tubule. Different results regarding the effect of angiotensin II on bicarbonate reabsorption and proton secretion have been reported at the functional level, depending on the angiotensin II concentration and tubule segment studied. It is likely that interstitial angiotensin II is more important in regulating hemodynamic and transport functions than circulating angiotensin II. In proximal tubules, stimulation of bicarbonate reabsorption, Na^+^/H^+^-exchange, and Na^+^/HCO_3_^–^ cotransport has been found using low concentrations (<10^–9^*M*), while inhibition of bicarbonate reabsorption has been documented using concentrations higher than 10^–8^*M*. Evidence for the regulation of H^+^-ATPase activity *in vivo* and *in vitro* by trafficking/exocytosis has been provided. An additional level of H^+^-ATPase regulation via protein synthesis may be important as well. Recently, we have shown that both aldosterone and angiotensin II provide such a mechanism of regulation *in vivo* at the level of the medullary collecting tubule. Interestingly, in this part of the nephron, the effects of aldosterone and angiotensin II are not sodium dependent, whereas in the cortical collecting duct, both aldosterone and angiotensin II, by contrast, affect H^+^ secretion by sodium-dependent mechanisms.

## INTRODUCTION

A main function of angiotensin II is the maintenance of extracellular $Na^+$ and volume homeostasis by controlling renal $Na^+$ reabsorption through the stimulation of $Na^+$-reabsorptive transport via the $Na^+/H^+$ antiporter, the epithelial $Na^+$-channel, and $Na^+/K^+$-ATPases[1,2,3,4]. Angiotensin II is a potent vasoconstrictor and, in addition to its effects on $Na^+$-homeostasis and blood pressure regulation, plays a role in the regulation of acid-base balance. Volume depletion leads to activation of angiotensin II, often resulting in metabolic alkalosis, suggesting a close association between the regulation of renal $Na^+$ and $HCO_3^-$ handling. Some of these effects are direct and others indirect via activation of aldosterone secretion by the adrenal gland. In the kidney, there is increasing evidence of a regulation of bicarbonate reabsorption and vacuolar $H^+$-ATPase activity by angiotensin II, as will be reviewed below.

## ANGIOTENSIN II

Angiotensin II is mainly formed by renin-mediated activation of circulating angiotensinogen, originating from the liver and after activation of angiotensin I by angiotensin converting enzyme (ACE). Intrarenal angiotensin II is regulated by several complex processes, involving formation of both systemically delivered and intrarenally formed substrate, as well as receptor-mediated internalization. Angiotensinogen, renin, and ACEs are also present along the nephron. Angiotensinogen is secreted into the lumen and activated there, forming a paracrine renin-angiotensin II system along the nephron[5,6]. The complex and extensive actions of angiotensin II on renal function are due to the widespread distribution of angiotensin II in nephron segments as well as in the vasculature and interstitium. Angiotensin II signals through two main receptor subtypes, $AT_1$ and $AT_2$[1,7,8]. Most actions of angiotensin II are primarily attributed to the $AT_1$ receptor because of its multiple vascular and transport effects. The $AT_1$ receptors have been localized in glomerular podocyte cells, proximal tubule brush border and basolateral membranes, thick ascending limb epithelia, distal tubule collecting ducts, macula densa cells, and interstitial cells of the renal medulla[9,10]. Due to the extensive localization of $AT_1$ receptors in luminal as well as basolateral membranes of proximal and distal nephron segments, interest is growing on the effects of angiotensin II at the tubular level.

Stimulation of the $AT_1$ receptor leads to increased $Na^+$, $HCO_3^-$, and fluid reabsorption along the nephron, which together with the induced vasoconstriction, result in a rise of blood pressure[1,11,12,13]. For these short-term effects, concentrations of angiotensin II in the picomolar range are needed, which is in contrast to higher nanomolar concentrations needed to activate the $AT_2$ receptor[1,14]. $AT_2$ receptor immunostaining in adult kidneys has been found in proximal tubules, collecting ducts, and renal vasculature. The $AT_2$ receptor seems to counterbalance the effects of the $AT_1$ receptor on electrolyte transport and blood pressure[14]. The very high abundance of $AT_1$ receptors in the kidney is associated with tissue angiotensin II levels that are much higher than plasma levels[15,16].

The intrarenal content of angiotensin II is not distributed in a homogenous manner, but is compartmentalized in both a regional and segmental manner[7]. Angiotensin II levels in the deep medulla are much higher than in the cortex and may be involved in the regulation of medullary hemodynamics[17,18]. Moreover, receptor binding studies have demonstrated that angiotensin II receptor density is much higher in the medulla than in the cortex[19]. Within the cortex, there is a substantial compartmentalization of intrarenal angiotensin II, with levels in the renal interstitial fluid and in proximal tubule fluid being much higher than that which can be accounted for by the circulating levels[20]. It is likely that interstitial angiotensin II is more important in regulating hemodynamic and transport functions than circulating angiotensin II. Angiotensin II in interstitial fluid might be responsible for maintaining tone of the pre- and postglomerular vessels and influencing tubular transport function by acting on angiotensin II receptors on the basolateral membranes of the tubular cells[21].

The finding that angiotensin II concentrations in perfused tubules were similar to those measured in nonperfused tubules indicates that the tubular fluid angiotensin II concentration is not derived from glomerular filtration[22]. Moreover, it has also been demonstrated that extracellular fluid volume expansion reduced plasma angiotensin II levels, but did not reduce intratubular angiotensin II concentrations[23].

Because angiotensin I and angiotensinogen are present in the proximal tubule fluid, intratubular angiotensin II could be formed from precursors secreted into the tubular lumen. The localization of intrarenal angiotensinogen mRNA and protein in proximal tubule cells indicates that proximal tubule cells provide the substrate for intratubular and interstitial angiotensin I and angiotensin II, and may not be dependent on circulating angiotensin II[24]. The angiotensinogen produced in proximal tubules is secreted into the tubular lumen. Angiotensin I may be formed in the tubular lumen by renin or renin-like enzymes[25]. In addition, cultured proximal cells have been found to produce renin and contain renin mRNA, thus suggesting that constitutive renin secretion occurs in proximal tubule cells[26]. When angiotensin I is formed, the conversion to angiotensin II occurs by the action of ACE in the proximal tubule brush border[27]. A novel ACE homologue, ACE 2, is also abundantly present in the proximal tubule and may play a counter-regulatory role by preventing angiotensin II accumulation[28]. ACE has been measured in proximal and distal tubular fluid, but is more abundant in proximal tubule fluid[29]. It seems that angiotensin II would have to be continuously produced or added to the proximal tubule fluid in view of the abundance of angiotensinases found in the brush border of proximal tubules[30]. It remains uncertain how much of the peptides are formed intracellularly and how much are formed in the tubular fluid.

The augmentation of intrarenal angiotensin II that occurs during physiologic increases in angiotensin levels includes a component of $AT_1$ receptor-mediated accumulation of angiotensin II into an intracellular compartment. Angiotensin peptides, ACE, and angiotensin II are present in renal endosomes[31]. The role of the internalized angiotensin II remains unclear. Angiotensin II might exert cytosolic actions. In addition, it has been suggested that angiotensin II migrates to the nucleus to exert transcriptional effects[32,33]. It has been shown that endocytosis of the angiotensin II/$AT_1$ receptor coupled to the activation of signal transduction pathways leads to enhanced sodium transport[34].

Previous studies support a role for luminal angiotensin II in regulating reabsorptive function in distal nephron and collecting ducts as well as in proximal tubule segments[12,35,36]. A direct relationship between urinary angiotensinogen excretion rates and renal angiotensin II has been demonstrated[37].

## REGULATION OF BICARBONATE REABSORPTION BY ANGIOTENSIN II IN PROXIMAL TUBULES

Several studies have shown, either in animal or cell culture models, that angiotensin II is a potent activator of bicarbonate reabsorption and proton secretory mechanisms along the nephron, thus stimulating overall bicarbonate reabsorption. The target transport mechanisms include the $Na^+/HCO_3^-$ cotransporter and $Na^+/H^+$ exchanger (at least NHE-3) in the proximal tubule, and the vacuolar $H^+$-ATPase in the proximal tubule, the distal convoluted tubule, and intercalated cells of the cortical collecting duct[12,38,39,40,41].

The proximal tubule is the primary site of $HCO_3^-$ reabsorption in the mammalian kidney. Angiotensin II plays an important role in modulating proximal tubule transport. Systemic infusion of angiotensin II, at doses that do not affect glomerular hemodynamics or blood pressure, result in a significant increase in proximal tubule transport. Both *in vivo* and *in vitro* microperfusion studies demonstrate that physiological concentrations of peritubular angiotensin II increase proximal tubule transport[42].

Recent studies have provided evidence for the local production and luminal secretion of angiotensin II by the proximal convoluted tubule (PCT), a mechanism that maintains a luminal concentration of angiotensin II along the proximal tubule higher than that measured in the systemic circulation[22,43]. The effect of luminal angiotensin II at low concentrations ($<10^{-9}$ *M*) increases $HCO_3^-$ reabsorption at 1.5-fold in the early segment of the proximal tubule[39]. This effect of angiotensin II is, at least in part, due to the stimulation of luminal $Na^+/H^+$ exchanger[12]. In this regard, angiotensin II, at the same low concentration ($10^{-9}$ *M*), has been shown to stimulate both an intracellular acid extruder (luminal $Na^+/H^+$ exchange) and an intracellular acid loader (basolateral $Na^+/HCO_3^-$ cotransport), thus greatly increasing transepithelial $HCO_3^-$ reabsorption in proximal tubules, with only a modest change in pHi[12].

Conversely, inhibition of bicarbonate reabsorption has been shown with angiotensin II concentrations higher than $10^{-8}$ *M*[44]. Stimulation of bicarbonate reabsorption, observed at low angiotensin II concentrations, has been attributable to inhibition of the production of cAMP via the G-protein $G_i$[45,46]. On the other hand, intracellular $Ca2^+$ serves as a second messenger in the inhibitory effect of high concentrations of angiotensin II on bicarbonate reabpsortion in the proximal tubule of the kidney[47].

Angiotensin II has potent effects on ammonia production and secretion rates by the proximal tubule. It has been demonstrated that isolated perfused mouse S3 proximal tubule segments produce and secrete ammonia. Angiotensin II added to the luminal fluid, enhanced renal ammonia production and secretion in S3 segments from acidotic mice[48].

## REGULATION OF BICARBONATE REABSORPTION BY ANGIOTENSIN II IN DISTAL TUBULE

Because relatively high concentrations of angiotensin II are present in the tubule fluid, it appears likely that this hormone might also have distinct effects on transport beyond the proximal tubule[13,40,49]. In distal tubules, the role of angiotensin II on acid secretion and bicarbonate reabsorption has been less extensively studied. Most of the previous studies evaluating electrolyte transport in the cortical distal tubule (DT) were based on either free-flow micropuncture or by perfusion of the whole distal tubule[50]. Indeed, *in vivo* microperfusion studies of the distal tubule have shown that an intravenous infusion of angiotensin II enhances distal tubule net bicarbonate reabsorption in the normal rat[40]. Since the distal tubule of cortical nephrons consists of several segments with distinct cell types and transport modes[51], angiotensin II transport modulation was examined separately on microperfused early and late distal tubule segments. By separate perfusion of early and late segments of cortical distal tubule *in vivo* (early segments corresponding to distal convoluted tubule and late segments to connecting segment and initial collecting duct), it has been shown that angiotensin II at concentration of $10^{-11}$ *M* stimulated early distal bicarbonate reabsorption by stimulating the $Na^+/H^+$ exchanger. The late distal effect of angiotensin II ($10^{-11}$ *M*) was mostly dependent on amiloride-sensitive $Na^+$ reabsorption, i.e., on sodium channels[36]. In luminal perfusion studies, it has been shown that angiotensin II stimulates $Na^+/H^+$ exchange in both early and late distal tubule via activation of $AT_1$ receptors, and the vacuolar $H^+$-ATPase in the late distal tubule. In all situations where the pharmacological action was evaluated, stimulation occurred through $AT_1$ receptors[35,49,52]. Consistent with these observations, Geibel et al. recently found that angiotensin II ($10^{-8}$ *M*) activates $H^+$-ATPase in intercalated cells via an $AT_1$ receptor which stimulates trafficking of the $H^+$-ATPase into the membrane[38]. In addition to acute effects of angiotensin II on vacuolar $H^+$-ATPase activity, it has been shown that chronic $AT_1$ blockade of *in vivo* microperfused distal tubules from remaining nephrons in 2/3 nephrectomized rats reduced bicarbonate reabsorption in close association with the reduction of synthesis and insertion of apical $H^+$-ATPase[53,54].

$AT_1$ receptors couple intracellularly mainly to phospholipase C, $Ca^{2+}$, and the PKC pathway[55]**.** Thus, it could be speculated that most stimulatory effects of angiotensin II on the vacuolar $H^+$-ATPase should be mediated via these signal pathways, but also through activation of tyrosine kinases[56]. In a subclone of MDCK cells exhibiting some characteristics of intercalated cells, angiotensin II was found to increase intracellular $Ca^{2+}$ and stimulate vacuolar $H^+$-ATPase activity. Moreover, chelating intracellular $Ca^{2+}$ abolished the stimulatory effect of angiotensin II[57].

In contrast to the studies of $H^+$-ATPase activation, one study using permeabilized CCD segments showed a specific dose-dependent inhibitory effect on the vacuolar $H^+$-ATPase in the presence of angiotensin II[58]. Weiner et al. demonstrated that angiotensin II acts through a basolateral $AT_1$ receptor to stimulate outer CCD luminal alkalinization in ß-intercallated cells[59]. Moreover, angiotensin II has been found to inhibit $HCO_3^-$ reabsorption to a similar extent in the absence and presence of luminal furosemide, which indicates that inhibition occurs independent from the effects on net NaCl absorption and is likely mediated through regulation of apical membrane $Na^+/H^+$ exchange in medullary thick ascending limb (MTAL). Thus, the inhibition of $Na^+/H^+$ exchange activity by angiotensin II in MTAL is an effect opposite to its physiological action to stimulate apical $Na^+/H^+$ exchange and $HCO_3^-$ reabsorption in the proximal tubule and early distal tubule. Angiotensin II inhibited $HCO_3^-$ reabsorption in MTAL in a concentration of $10^{-8}$ *M*, similar to angiotensin II levels measured in the renal medulla *in vivo*. This inhibition is mediated by the cytochrome P-450–dependent signaling pathway. Tyrosine kinase pathways also appear to play a role in the angiotensin II–induced transport inhibition[60].

A reduction in bicarbonate reabsorption after application of angiotensin II was also found in perfused rat outer medullary collecting ducts[61]. This effect of angiotensin II was not observed in the presence of the $AT_1$ receptor blocker, candesartan. Although angiotensin II upregulates $H^+$ secretion in proximal and distal tubules, angiotensin II may reduce $H^+$ secretion in other segments when perfused *in vitro* depending on angiotensin II concentration and tubule segments studied. An inhibitory effect on distal $H^+$ excretion, however, would be unexpected in light of the stimulatory effect of angiotensin II on vacuolar $H^+$-ATPase (see below).

## VACUOLAR $H^+$-ATPASE

Vacuolar $H^+$-ATPases are ubiquitous multisubunit complexes mediating ATP-driven vectorial transport of protons across membranes. The vacuolar $H^+$-ATPases are hetero-oligomeric complexes composed of 13 polypeptides arranged into a soluble cytoplasmic $V_1$ domain composed of 8 subunit types, which is the site of ATPase activity, and a membrane-associated Vo domain composed of 5 subunit types that includes the proton translocation pathway[62,63,64,65]. Little is known about the expression patterns of these subunits in the kidney with the exception of the B1 and "a4" subunits. The B1 isoform is a part of the peripheral $V_1$ domain[62]. Two isoforms of the B subunit exist: the B2 isoform (ATP6V1B2) is ubiquitously expressed[63,64], whereas, in the kidney, the B1 subunit is present in intercalated cells of the late distal tubule, connecting segment, and cortical and medullary collecting ducts[65]. The B1 subunit is also expressed in the cochlea and endolymphatic sac[66]. The "a4" subunit (ATP6V0A4) is one of four isoforms of the "a" subunit which form part of the membrane bound $V_0$ sector. All four "a" subunits are expressed in the kidney as detected by Northern blot and RT-PCR analysis[67,68,69]. The "a4" isoform is expressed in the kidney (proximal tubule, loop of Henle, and all subtypes of intercalated cells along the late distal tubule, connecting segment, and entire collecting duct)[70].

Mutations in either the B1 or the 116-kDa subunit (a4 isoform) cause inherited forms of distal renal tubular acidosis (dRTA). Whereas mutations in the B1 subunit (ATP6B1) cause dRTA with sensorineural deafness[66], mutations in the 116-kD accessory subunit (ATP6N1B) affect predominantly the kidney[71,72].

## ANGIOTENSIN II REGULATION OF VACUOLAR $H^+$-ATPASE ACTIVITY BY TRAFFICKING/EXOCYTOSIS

Regulation of $H^+$-ATPase activity includes mechanisms such as trafficking from submembraneous pools, regulation by cytosolic activator and inhibitor proteins, or gene expression and protein expression[73]. There is evidence for regulation of $H^+$-ATPase activity *in vivo* and *in vitro* by trafficking/exocytosis.

The B1 subunit interacts directly with filamentous actin, which could allow for the trafficking[74]. Recently, in cell culture, evidence has been found for the involvement of the vesicular SNARE docking proteins in the fusion of $H^+$-ATPase containing vesicles with the plasma membrane[75,76]. Moreover, the B1 subunit of the kidney $H^+$-V-ATPase has been identified as a PDZ domain-binding protein that colocalized with NHE-RF in renal type B-intercalated cells, providing a target for regulation. NHE-RF has been shown to link other transport proteins to the cytoskeleton and may have a role in the regulation of $H^+$-ATPase localization or trafficking in B intercalated cells[77].

Regulation of a4 isoform subunit by trafficking, but not protein expression, with a marked redistribution of the subunit in intercalated cells in response to acid-base changes and dietary potassium intake, has recently been reported[70]. These findings are in good agreement with earlier reports showing trafficking of other $H^+$-ATPase subunits in response to metabolic acidosis or alkalosis or dietary electrolyte intake [78]. In intercalated cells of isolated mouse cortical collecting ducts, angiotensin II ($10^{-8}$ *M*) stimulation of vacuolar $H^+$-ATPase has been shown to be prevented by colchicine, an agent that disrupts the microtubular network. This suggests that vacuolar $H^+$-ATPases, some of their subunits, or other stimulatory proteins may be trafficked to the membrane[38].

The electrogenic vacuolar $H^+$-ATPase, by the translocation of $H^+$ ions across membranes, renders the cell interior negative with respect to the exterior. This process generates both a transmembrane potential and a chemical gradient, resulting in a self-limiting activity of the vacuolar $H^+$-ATPase[79]. In many intracellular organelles and at cell plasma membranes, a parallel $Cl^-$ conductance provides an electric shunt, compensating for the positive charge transferred by the pump and, thus, dissipating the electrical gradient[80,81,82,83].

The presence of this parallel $Cl^-$ conductance has been found in most intracellular organelles that are acidified by vacuolar $H^+$-ATPases, such as lysosomes[84] and endosomes[80,81,85]. In the kidney, it is clear that vacuolar $H^+$-ATPase–dependent acidification is $Cl^-$ dependent in endosomal fractions[80,81,85,86,87,88] and in brush border membrane vesicles[89,90]. The chloride dependence on plasma membrane $H^+$-ATPase, however, is a complex issue because of the expression of numerous other conductances or electrogenic transporters in apical and basolateral membrane domains[79]. In isolated rat proximal tubules, vacuolar $H^+$-ATPase–dependent $H^+$ extrusion was reduced after preincubation in $Cl^-$-free media[91]. In another study, $Na^+$-independent, $H^+$-ion secretion was stimulated by physiological concentration of angiotensin II. This angiotensin II stimulation of $H^+$-ATPase–dependent proton extrusion was demonstrated to occur via a process involving a $Cl^-$-dependent insertion of vesicles into the brush border membrane[41]. In addition, in mouse proximal tubules, the insertion of vacuolar $H^+$-ATPase containing vesicles was delayed in the absence of chloride[92]. Notwithstanding, there has not been convincing evidence of the role of chloride in apical $H^+$-ATPase–dependent proton secretion in intercalated cells. The *in vivo* microperfusion of late distal tubule, however, showed a reduction of electrogenic $H^+$ secretion by the $Cl^-$ channel blocker, NPPB, indicating coupling between $H^+$-ATPase and $Cl^-$ transport[93].

Bicarbonate reabsorption is independent of the presence of luminal chloride in isolated rabbit medullary collecting duct, although it is completely abolished in the absence of basolateral chloride, presumably due to impairment of peritubular bicarbonate exit in exchange for $Cl^-$ via the $Cl^-/HCO_3^-$ exchanger AE1[94].

## ANGIOTENSIN II AND EXPRESSION OF VACUOLAR $H^+$-ATPase SUBUNITS *IN VIVO*

It has been generally believed that $H^+$-ATPase is regulated to only a minor extent by transcriptional and translational mechanisms and very little is known about the *in vivo* regulation of the various subunits of the $H^+$-ATPase. Recently, we examined the expression of $H^+$-ATPase subunits, B1 and a4, in an animal model of selective aldosterone deficiency (adrenalectomized [ADX] rats maintained on glucocorticoid replacement). The effect of exogenous angiotensin II on the *in vivo* expression of the B1 $H^+$-ATPase subunit was also studied[95].

Our data showed an increase in the relative protein abundance of B1 subunit isoform of the $H^+$-ATPase after chronic infusion of angiotensin II in the renal medulla of ADX rats. This increase in $H^+$-ATPase expression by angiotensin II was limited to intercalated cells in the medullary collecting tubule. The antibody to the kidney isoform of the B1 subunit stained intensely on apical membrane of intercalated cells from outer medullary collecting tubules and initial inner medullary collecting tubules in ADX infused with angiotensin II. By contrast, we did not find significant differences in B1 subunit of $H^+$-ATPase protein in cortical membrane preparations. The lack of difference in B1 protein expression renal cortex could reflect, in part, the predominance of renal proximal tubules, which do not express B1. In contrast to the B1 subunit, the expression of the a4 subunit was not altered significantly by either ADX or exogenous angiotensin II addition. Interestingly, the a4 subunit is found in both the early segments of proximal tubules and the intercalated cells in the kidney. The B1 subunit of the kidney $H^+$-V-ATPase, however, is expressed only in the intercalated cells of the kidney collecting tubules. Thus, one could speculate that regulation of the B1 subunit could be a mechanism by which hormones, such as aldosterone and angiotensin II, specifically control $H^+$-ATPase synthesis in intercalated cells of the distal nephron.

Related to the different effect of angiotensin II and ADX on medullary and cortical collecting tubules, it has been previously demonstrated that medullary angiotensin II levels are higher than the cortical levels in normal rats and increase further in angiotensin II–infused hypertensive rats[96]. In addition, increased uptake and trafficking of angiotensin II into renal endosomes is mediated by $AT_1$ receptors in renal cortex after chronic angiotensin II infusion[97]. This could suggest that in renal cortex, B1 regulation is under control of a mechanism(s) other than protein synthesis. Regulation of a4 and B1 subunits by trafficking, but not protein expression, has been demonstrated under conditions of acid base and electrolyte changes[38,41,67]. It should be noted that different results regarding the effect of angiotensin II on $H^+$ secretion have been reported at the functional level depending on the angiotensin II concentration and tubule segments studied[12,54].

On a functional level, aldosterone stimulates electrogenic $Na^+$ reabsorption through the luminal epithelial $Na^+$ channel resulting in lumen-negative potential that leads to increase the electrical driving force for $H^+$ secretion in the cortical collecting duct[98,99]. A direct action of aldosterone on $H^+$ secretion has been shown in rat medullary collecting duct. The effect of aldosterone was independent of $Na^+$ in medullary collecting ducts perfused *in vitro* pointing also to mechanisms in addition to changes in the electrical driving force[100]. Different sensitivities of vacuolar $H^+$-ATPase enzymatic activity in distinct collecting duct segments have been described, even though aldosterone stimulated $H^+$-ATPase–dependent bicarbonate reabsorption in all collecting duct segments[101,102].

Our findings in ADX rats suggest that the removal of aldosterone results in a decrease in the relative abundance and expression of B1 $H^+$-ATPase in the medullary, but not the cortical, collecting tubule. These results suggest that aldosterone affects vacuolar $H^+$-ATPase activity in the collecting duct through different mechanisms that may be specific for tubular segment and cell type. Our data suggest that some of aldosterone actions may require protein synthesis of $H^+$-ATPase subunits.

We also have found that aldosterone increases a4 $H^+$-ATPase synthesis in a cell line of the collecting duct[103]. Importantly, in microdissected cortical and medullary collecting duct segments from ADX rats, a pharmacological dose of aldosterone increased enzyme $H^+$-ATPase activity irrespective of potassium level[104]. A recent study showed nongenomic targeting of the a4 subunit of vacuolar $H^+$-ATPase to the apical membrane by aldosterone[105].

An additional level of regulation via protein synthesis, however, may be important as well. Our results suggest that both aldosterone and angiotensin II provide such a mechanism of regulation *in vivo* at the level of the medullary collecting tubule. Interestingly, in this part of the nephron, the effects of aldosterone and angiotensin II are not sodium dependent whereas in the cortical collecting duct both aldosterone and angiotensin II, by contrast, affect $H^+$ secretion by sodium-dependent mechanisms[98,100].

In summary, angiotensin II and ADX affect $H^+$-ATPase B1-subunit protein expression in medullary collecting ducts[95]. Selective aldosterone deficiency created by adrenalectomy with glucocorticoid replacement resulted in the downregulation of the expression of $H^+$-ATPase B1 subunit in medullary collecting ducts. Angiotensin II increased the expression of the B1 subunit of $H^+$-ATPase in the medullary collecting duct and thus may upregulate $H^+$ secretion in this tubule segment. Such an action may be responsible, in part, for an increase in $H^+$ secretion, independent of aldosterone[95]. The expression of the a4 subunit, by contrast, was not altered by either ADX or exogenous angiotensin II[95]. Angiotensin II exerts significant direct effects on acid-base balance, separate from those related to its stimulatory effect on aldosterone secretion. The renal vacuolar $H^+$-ATPase is an important target for angiotensin II action particularly in renal medullary collecting tubules.
